# Pathways to reduced overnight hospitalizations in older adults: Evaluating 62 physical, behavioral, and psychosocial factors

**DOI:** 10.1371/journal.pone.0277222

**Published:** 2022-11-10

**Authors:** Julia S. Nakamura, Jean Oh, Tyler J. VanderWeele, Eric S. Kim

**Affiliations:** 1 Department of Psychology, University of British Columbia, Vancouver, British Columbia, Canada; 2 Human Flourishing Program, Institute for Quantitative Social Science, Harvard University, Cambridge, Massachusetts, United States of America; 3 Department of Biostatistics, Harvard T.H. Chan School of Public Health, Harvard University, Boston, Massachusetts, United States of America; 4 Department of Epidemiology, Harvard T.H. Chan School of Public Health, Harvard University, Boston, Massachusetts, United States of America; 5 Department of Social & Behavioral Sciences, Harvard T.H. Chan School of Public Health, Harvard University, Boston, Massachusetts, United States of America; 6 Lee Kum Sheung Center for Health and Happiness, Harvard T.H. Chan School of Public Health, Harvard University, Boston, Massachusetts, United States of America; Universitatsklinikum Schleswig Holstein Campus Lubeck, GERMANY

## Abstract

As our society ages and healthcare costs escalate, researchers and policymakers urgently seek potentially modifiable predictors of reduced healthcare utilization. We aimed to determine whether changes in 62 candidate predictors were associated with reduced frequency, and duration, of overnight hospitalizations. We used data from 11,374 participants in the Health and Retirement Study—a national sample of adults aged >50 in the United States. Using generalized linear regression models with a lagged exposure-wide approach, we evaluated if changes in 62 predictors over four years (between t_0_;2006/2008 and t_1_;2010/2012) were associated with subsequent hospitalizations during the two years prior to t_2_ (2012–2014 (Cohort A) or 2014–2016 (Cohort B)). After robust covariate-adjustment, we observed that changes in some health behaviors (e.g., those engaging in frequent physical activity had 0.80 the rate of overnight hospital stays (95% CI [0.74, 0.87])), physical health conditions (e.g., those with cancer had 1.57 the rate of overnight hospital stays (95% CI [1.35, 1.82])), and psychosocial factors (e.g., those who helped friends/neighbors/relatives 100–199 hours/year had 0.73 the rate of overnight hospital stays (95% CI [0.63, 0.85])) were associated with subsequent hospitalizations. Findings for both the frequency, and duration, of hospitalizations were mostly similar. Changes in a number of diverse factors were associated with decreased frequency, and duration, of overnight hospitalizations. Notably, some psychosocial factors (e.g., informal helping) had effect sizes equivalent to or larger than some physical health conditions (e.g., diabetes) and health behaviors (e.g., smoking). These psychosocial factors are mostly modifiable and with further research could be novel intervention targets for reducing hospitalizations.

## Introduction

Three factors converge to underscore the heightening importance of identifying factors that reduce the frequency, and duration, of overnight hospital stays. First, is our rapidly aging population. There are 46.3 million people aged >65 years in the United States and in the next 15 years, this age-group is projected to increase by nearly 50% [1]. Second is the rising costs of medical care. The Centers for Medicare and Medicaid Services projects that national health expenditures will nearly double from $3.6 trillion in 2018 to $6.2 trillion in 2028 [2], and hospitalizations are a major driver of costs. Despite composing <15% of the population, people aged ≥65 account for 31% of hospital care spending [2, 3]. Third, growing evidence documents how hospitalizations place patients at elevated risk for hospital-acquired infections (e.g., multidrug resistant infections) and other unintended consequences resulting from hospital-associated stressors (e.g., sleep deprivation, social isolation, and poor nutrition) [4]. In concert, these three factors generate substantial psychological, physical, and financial burden to: individuals, healthcare systems, and governments. Thus, a central challenge is to identify factors that reduce the frequency, and duration, of overnight hospital stays.

Several factors have been evaluated as predictors of overnight hospital stays. The majority of literature has focused on and observed that physical health conditions (e.g., hypertension, cancer, diabetes, heart disease, stroke, cognitive impairment [5–8]) and unhealthy behaviors (e.g., insomnia, physical inactivity, poor diet, smoking, excessive alcohol consumption [9–11]) increase hospitalizations. Fewer studies have evaluated psychosocial factors. Existing evidence suggests that psychological well-being factors (e.g., purpose in life, health and financial mastery, positive affect, and self-efficacy [12–14]) decrease hospitalizations, while psychological distress factors (e.g., negative affect, depressive symptoms) increase hospitalizations [13, 15]. Further, social factors (e.g., volunteering, living alone, social connections, support, and participation, social network size [16–21]) have also been associated with subsequent hospitalizations. However, some of these psychosocial predictors have mixed results. For example, in some studies, marital status is not linked with hospitalizations [17], in others, being single or divorced is associated with increased risk of hospitalizations [21, 22], and still in other studies being unmarried is associated with decreased risk of hospitalizations [18]. Further, to our knowledge, some psychosocial factors have not been evaluated at all (e.g., hopelessness, daily discrimination) in the context of hospitalizations. There are many plausible pathways connecting psychosocial factors with hospitalizations. For example, enhanced purpose in life may lead to a greater will to live, which could help people endure short-term discomfort in exchange for longer-term rewards [23]. This might translate to a higher likelihood of engaging in health-preventive behaviors, which may decrease subsequent hospitalizations. Indeed, one recent study suggests that when confronted with competing decisions (e.g., “should I take the stairs or elevator?”), people with higher purpose experience increased receptivity to health advice [24]. As another example, the association between stronger social support and increased adherence to medical treatment may be the mechanism explaining decreased subsequent hospitalizations [25].

These pioneering studies have contributed substantially to the literature but remain limited in several ways. First, many studies were conducted in small samples with specific subpopulations (e.g., heart failure patients, low-income public rental flat dwellers), and results might not generalize [26, 27]. Second, many studies inadequately adjusted for potential confounders. Third, while a sizable portion of existing studies are longitudinal, many are cross-sectional, and we are unable to establish directionality [27, 28]. Fourth, most longitudinal studies do not adjust for predictors in the pre-baseline wave (nor, pre-baseline overnight hospitalizations), and doing so allows researchers to ask a slightly different question more aligned with the interests of interventionists and policy-makers: If a given predictor is modified, what change in overnight hospitalizations might we observe?

To begin addressing this question, we used a new lagged exposure-wide analytic approach (see Statistical analysis section) [29]. Using generalized linear regression models with a lagged exposure-wide approach, we evaluated if changes in 62 predictors over four years (between t_0_;2006/2008 and t_1_;2010/2012) were associated with subsequent hospitalizations during the two years prior to t_2_ (2012–2014 (Cohort A) or 2014–2016 (Cohort B)). This method allowed us to individually regress hospitalizations in the two years prior to the outcome wave on each of 62 baseline candidate predictors. Thus, we could prospectively test if changes in these predictors were associated with subsequent hospitalizations. This approach helped us assess a broad spectrum of potential antecedents of hospitalizations and compare effect sizes as we used the same population, study design, analytic methods, and covariates. Exposure-wide analyses are a hypothesis-generating, data-driven approach that can be used to discover promising predictors of overnight hospitalizations, which may then undergo further investigation in future studies. We chose these 62 predictors because: 1) they are frequently included in the conceptualization of key gerontological models that characterize the antecedents, processes, and outcomes that foster people’s ability to age well [30–34], and 2) most have shown evidence they can be modified, or are likely modifiable with further research.

The aim of our study was to highlight specific physical-, behavioral-, and psychosocial-factors associated with overnight hospitalizations in older adults. We found that changes in several candidate predictors were associated with decreased frequency and duration of overnight hospitalizations.

## Methods

### Study population

We used data from the Health and Retirement Study (HRS)–a national panel study of Americans aged >50. In 2006, when psychosocial data were first collected, half of HRS respondents completed an enhanced face-to-face interview. The other half were assessed in 2008. Participants then completed a psychosocial questionnaire which they mailed to the University of Michigan upon completion (response rates of 88% in 2006 and 84% in 2008) [35]. These sub-cohorts alternate reporting on psychosocial factors (each participant reports psychosocial data every 4 years). To increase sample size and statistical power, data from the 2006 and 2008 sub-cohorts were combined. Since >50% of study predictors were psychosocial factors, participants were excluded if they did not report psychosocial data in the pre-baseline wave. To keep the maximum number of total nights in the hospital and overnight hospital stays consistent across all participants, we excluded anyone that was not interviewed in the two years prior to t_2_ (2014/2016), resulting in a final sample of 11,374 participants.

This study used data from three time points. Covariates were assessed in the pre-baseline wave (t_0_;2006/2008), candidate predictors were assessed in the baseline wave (t_1_;2010/2012), and outcomes were assessed in the two years leading up to the outcome wave (t_2_;2014/2016). The HRS is coordinated by the University of Michigan and sponsored by the National Institute on Aging (NIA U01AG009740; see further HRS documentation at: http://hrsonline.isr.umich.edu/) [36].

### Measures

#### Hospitalizations

Respondents reported: 1) the number of overnight hospital stays they had since their prior interview (past 2 years for our sample), and 2) the total nights spent in the hospital across all hospital stays. Due to the notable costs of both separate hospital stays (ranging from $10,381 (people aged ≥95) to $14,072 (people aged 65–74)) and the added cost of each individual night (ranging from $2,133 (people aged ≥95) to $2,696 (people aged 65–74)), we evaluated both outcomes [37].

#### Covariates

We adjusted for a large number of covariates in the pre-baseline wave (t_0_;2006/2008), including: sociodemographic factors (age (continuous), gender (male/female), race/ethnicity (White, African-American, Hispanic, Other), marital status (married/not married), income (<$50,000, $50,000-$74,999, $75,000-$99,999, ≥$100,000), total wealth (based on quintiles of the score distribution for total wealth in this sample), educational attainment (no degree, GED/high school diploma, ≥college degree), employment status (yes/no), health insurance (yes/no), geographic region (Northeast, Midwest, South, West), religious service attendance (none, <1x/week, ≥1x/week), personality (openness, conscientiousness, extraversion, agreeableness, neuroticism; continuous), and childhood abuse (yes/no)). We also adjusted for: 1) prior values of all predictors to examine change in each predictor and 2) pre-baseline overnight hospitalizations to reduce the possibility of reverse causation. These covariates were selected as common causes of the exposure, the outcome, or of both [38] following recent epidemiological guidelines for covariate selection. A substantial number of covariates were selected with the ultimate goal being that, conditional on the final covariate set, the groups with and without a given predictor are comparable [29].

#### Predictors

We evaluated 62 candidate predictors in the baseline wave (t_2_:2010/2012), including measures of: 1) health behaviors (physical activity, smoking, heavy drinking, sleep problems); 2) physical health (number of physical conditions, diabetes, hypertension, stroke, cancer, heart disease, lung disease, arthritis, overweight/obesity, physical functioning limitations, cognitive impairment, chronic pain, self-rated health, hearing, eyesight); 3) psychological well-being (positive affect, life satisfaction, optimism, purpose in life, mastery, health mastery, financial mastery); 4) psychological distress (depression, depressive symptoms, hopelessness, negative affect, perceived constraints, anxiety, trait anger, state anger, cynical hostility, stressful life events, financial strain, daily discrimination, major discrimination); 5) social factors (living with a spouse/partner, frequency of contact in 3 separate relationship categories: (i) children, (ii) other family, and (iii) friends, loneliness, closeness with spouse, number of close (i) children, (ii) other family, and (iii) friends, positive social support from (i) spouse, (ii) children, (iii) other family, and (iv) friends, negative social strain from (i) spouse, (ii) children, (iii) other family, and (iv) friends, religious service attendance, volunteering, helping friends/neighbors/relatives, social status ladder ranking, and change in social status ladder ranking); and 6) work (in the labor force). S1 Appendix and the HRS Materials provide further details about each of these variables [35, 39, 40].

#### Multiple imputation

All missing exposures, covariates, and outcome variables were imputed using imputation by chained equations. This method is more flexible than other methods of handling missing data [41] and addresses problems that arise from attrition [42–44]. Ten datasets were created. Theoretical work suggests that adding more imputed datasets (e.g., m>5) does not typically change substantive conclusions of the analysis [45] and increasing the number of imputations will substantially increase computational burden. Though we originally used five imputed datasets (consistent with prior exposure-wide studies in HRS [46]), ten were required in this study for some supplemental analyses to converge with at least five imputed datasets being incorporated in analyses (see S1 Table footnote).

### Statistical analysis

Analyses were performed in Stata 17.0 (StataCorp LLC). We used an exposure-wide analytic approach [29] and ran separate models for each exposure. The total nights spent in the hospital and the number of overnight hospital stays were both count outcomes. Thus, we used generalized linear models (with a log link and a negative binomial distribution) to individually regress hospitalizations in the two years prior to the outcome wave (t_2_:2014/2016) on baseline (t_1_;2010/2012) candidate predictors, as these models were the most efficient and correctly specified. All continuous predictors were standardized (mean = 0, standard deviation = 1) so their effect sizes could be interpreted as a standard deviation change in the exposure variable. For categorical exposures, the effect estimate corresponds to associations between the exposures at baseline (t_1_;2010/2012) and hospitalizations in the two years prior to the outcome wave (t_2_:2014/2016), conditional on the exposures in the pre-baseline wave (t_0_;2006/2008; see S2 Appendix for a proof illustrating how adjusting for pre-baseline levels of exposure variables helps us evaluate associations between “changes” in exposures and our outcomes of interest). We marked multiple p-value cutoffs (including Bonferroni-corrected) and gave exact confidence intervals in our tables because multiple testing practices vary widely and are continuously evolving [47, 48].

#### Additional analyses

We conducted several additional analyses. First, because one can never be certain that the assessed covariates suffice to adjust for confounding or that any remaining unmeasured confounding is small, sensitivity analyses for unmeasured confounding and other biases are important [29]. Thus, we conducted E-value analyses to assess the minimum strength of unmeasured confounding associations on the risk ratio (RR) scale (with both the exposure and the outcome) needed to explain away the association between the exposure and outcome so as to evaluate the robustness of our results to potential unmeasured confounding [49]. Second, because our overnight hospitalization variables exhibited overdispersion and excess zeros (i.e., a large number of individuals who did not have any hospitalizations), we repeated our analyses using zero-inflated negative binomial regression (S1 and S2 Tables) and zero-inflated Poisson regression (S3 and S4 Tables). Zero-inflated models are designed to handle situations with an “excessive” number of individuals that have responses of zero, and these analyses result in two types of models. The first model is a negative binomial (S1 and S2 Tables) or Poisson (S3 and S4 Tables) model that predicts respondents who are “not certain zeros” (i.e., respondents who are not always certain to have values of zero for hospitalizations variables). The second model is a logistic model that predicts respondents who are “certain zeros” (i.e., respondents who always have values of zero for hospitalizations). Third, we repeated our main analyses using only complete-cases to assess the impact of multiple imputation on results (S5 and S6 Tables).

### Ethical issues/statement

This study was exempt from review by the ethics board at the University of British Columbia because it used de-identified and publicly available data. All respondents provided written informed consent to the Health and Retirement Study. Additional information about the HRS can be found on their website: http://hrsonline.isr.umich.edu.

## Results

In the pre-baseline wave (t_0_;2006/2008), participants were on average 68 years old (SD = 9), predominantly women (59%) and married (64%). Table 1 provides the distribution of covariates in the pre-baseline wave.

**Table 1:** Characteristics of participants at pre-baseline (N = 11,374)^a^^,^^b^.

Participant Characteristics	No. (%)	Mean (SD)
***Sociodemographic Factors***		
Age (yr; range: 52–104)		68.3 (9.2)
Female (%)	6764 (59.5)	
Race/ethnicity (%)		
White	8786 (77.3)	
Black	1438 (12.6)	
Hispanic	911 (8.0)	
Other	238 (2.1)	
Married (%)	7260 (63.8)	
Annual household income (%)		
<$50,000	6683 (58.8)	
$50,000-$74,999	1818 (16.0)	
$75,000-$99,999	1020 (9.0)	
≥$100,000	1853 (16.3)	
Total wealth (%)		
1st Quintile	2281 (20.1)	
2nd Quintile	2274 (20.0)	
3rd Quintile	2279 (20.0)	
4th Quintile	2272 (20.0)	
5th Quintile	2268 (19.9)	
Education (%)		
<High school	2063 (18.2)	
High school	6270 (55.3)	
≥College	3014 (26.6)	
Employment		
In labor force	4304 (37.8)	
Health insurance (%)	10875 (95.7)	
Geographic region (%)		
Northeast	1683 (14.8)	
Midwest	3059 (26.9)	
South	4493 (39.6)	
West	2121 (18.7)	
Childhood abuse (%)	731 (6.5)	
***Physical Health***		
Number of physical conditions (range: 0–8)		2.5 (1.4)
Diabetes (%)	2108 (18.6)	
Hypertension (%)	6338 (55.8)	
Stroke (%)	772 (6.8)	
Cancer (%)	1595 (14.1)	
Heart disease (%)	2471 (21.7)	
Lung disease (%)	918 (8.1)	
Arthritis (%)	6739 (59.3)	
Overweight/obesity (%)	8037 (71.5)	
Physical functioning limitations (%)	2430 (21.4)	
Cognitive impairment (%)	1933 (17.2)	
Chronic pain (%)	3864 (34.0)	
Self-rated health (range: 1–5)		3.2 (1.1)
Hearing (range: 1–5)		3.3 (1.1)
Eyesight (range: 1–6)		4.2 (1.0)
***Health Behaviors***		
Heavy drinking (%)	664 (7.2)	
Smoking (%)	1361 (12.1)	
Frequent physical activity (%)	8478 (74.6)	
Sleep problems (%)	2518 (41.2)	
***Psychological Well-Being***		
Positive affect (range: 1–5)		3.6 (0.7)
Life satisfaction (range: 1–7)		5.1 (1.5)
Optimism (range: 1–6)		4.5 (1.0)
Purpose in life (range: 1–6)		4.6 (0.9)
Mastery (range: 1–6)		4.8 (1.1)
Health mastery (range: 0–10)		7.3 (2.3)
Financial mastery (range: 0–10)		7.4 (2.6)
***Psychological Distress***		
Depression (%)	1467 (13.1)	
Depressive symptoms (range: 0–8)		1.3 (1.9)
Hopelessness (range: 1–6)		2.3 (1.3)
Negative affect (range: 1–5)		1.7 (0.6)
Constraints (range: 1–6)		2.2 (1.2)
Anxiety (range: 1–4)		1.6 (0.6)
Trait anger (range: 1–4)		2.2 (0.7)
State anger (range: 1–4)		1.5 (0.5)
Cynical hostility (range: 1–6)		2.9 (1.1)
Stressful life events (range: 0–5)		0.2 (0.6)
Financial strain (range: 1–5)		2.0 (1.0)
Daily discrimination (range: 1–6)		1.6 (0.7)
Major discrimination (range: 0–6)		0.5 (0.9)
***Social Factors***		
Living with spouse/partner (%)	7432 (67.1)	
Contact children (%)		
<Every few months	1468 (13.2)	
1-2x/month	1255 (11.3)	
1-2x/week	3490 (31.5)	
≥3x/week	4878 (44.0)	
Contact other family (%)		
<Every few months	2662 (23.9)	
1-2x/month	2624 (23.6)	
1-2x/week	3094 (27.8)	
≥3x/week	2739 (24.6)	
Contact friends (%)		
<Every few months	1769 (15.8)	
1-2x/month	2063 (18.5)	
1-2x/week	4066 (36.4)	
≥3x/week	3283 (29.4)	
Loneliness (range: 1–3)		1.5 (0.5)
Closeness with spouse (range: 1–4)		3.5 (0.7)
Number of close children		2.8 (3.7)
Number of close other family		3.9 (5.5)
Number of close friends		4.5 (5.9)
Positive social support from spouse (range: 1–4)		3.5 (0.7)
Positive social support from children (range: 1–4)		3.3 (0.7)
Positive social support from other family (range: 1–4)		2.9 (0.9)
Positive social support from friends (range: 1–4)		3.1 (0.7)
Social strain from spouse (range: 1–4)		2.0 (0.7)
Social strain from children (range: 1–4)		1.7 (0.6)
Social strain from other family (range: 1–4)		1.6 (0.6)
Social strain from friends (range: 1–4)		1.8 (0.4)
Religious service attendance (%)		
Not at all	2685 (23.6)	
<1x/week	3591 (31.6)	
≥1x/week	5091 (44.8)	
Volunteering (%)		
0 hours/year	7107 (62.6)	
1–49 hours/year	1353 (11.9)	
50–99 hours/year	952 (8.4)	
100–199 hours/year	1044 (9.2)	
≥200 hours/year	905 (8.0)	
Helping friends/neighbors/relatives (%)		
0 hours/year	5143 (45.3)	
1–49 hours/year	2779 (24.5)	
50–99 hours/year	1614 (14.2)	
100–199 hours/year	1049 (9.3)	
≥200 hours/year	758 (6.7)	
Social status ladder (range: 1–10)		6.6 (1.7)
Change in social status ladder (%)		
Moved down	1058 (9.6)	
No change	8494 (77.4)	
Moved up	1428 (13.0)	
***Personality***		
Openness (range: 1–4)		2.9 (0.5)
Conscientiousness (range: 1–4)		3.4 (0.5)
Extraversion (range: 1–4)		3.2 (0.5)
Agreeableness (range: 1–4)		3.5 (0.5)
Neuroticism (range: 1–4)		2.0 (0.6)

Abbreviations: SD, standard deviation.

^a^This table was created based on non-imputed data.

^b^All variables in Table 1 were used as covariates, and assessed in the pre-baseline wave (t_0_;2006/2008).

S7 Table describes changes in hospitalizations from the pre-baseline (t_0_) to the outcome wave (t_2_). Results for both sets of outcomes were largely similar. Thus, we focused on results evaluating the number of separate overnight hospital stays and mentioned results from the total nights spent in the hospital whenever there were notable divergences. Tables 2 and 3 show associations between candidate predictors and both outcomes.

**Table 2:** Candidate predictors of overnight hospital stays (Health and Retirement Study [HRS]: N = 11,374)^a^^,^^b^^,^^c^.

Candidate Predictor	Rate Ratio	95% CI
**Health Behaviors**		
Frequent physical activity	0.80	0.74, 0.87***
Smoking	0.77	0.63, 0.93**
Heavy drinking	0.99	0.77, 1.27
Sleep problems	1.10	0.95, 1.26
**Physical Health**		
Number of physical conditions	1.36	1.27, 1.46***
Diabetes	1.17	1.02, 1.34*
Hypertension	1.13	0.98, 1.29
Stroke	1.29	1.11, 1.50**
Cancer	1.57	1.35, 1.82***
Heart disease	1.48	1.32, 1.67***
Lung disease	1.56	1.33, 1.83***
Arthritis	1.23	1.04, 1.46*
Overweight/obese	0.99	0.89, 1.11
Physical functioning limitations	1.40	1.29, 1.53***
Cognitive impairment	1.05	0.95, 1.16
Chronic pain	1.15	1.06, 1.25***
Self-rated health	0.77	0.73, 0.80***
Hearing	0.96	0.91, 1.01
Eyesight	0.93	0.90, 0.97***
**Psychological Well-being**		
Positive affect	0.96	0.91, 1.01
Life satisfaction	0.91	0.87, 0.96***
Optimism	0.99	0.95, 1.03
Purpose in life	0.92	0.87, 0.98*
Mastery	0.92	0.88, 0.97**
Health mastery	0.92	0.88, 0.96***
Financial mastery	0.99	0.95, 1.03
**Psychological Distress**		
Depression	1.08	0.96, 1.20
Depressive symptoms	1.08	1.03, 1.13***
Hopelessness	1.00	0.95, 1.05
Negative affect	1.10	1.06, 1.15***
Constraints	1.17	1.12, 1.23***
Anxiety	1.15	1.08, 1.23***
Trait anger	0.98	0.94, 1.02
State anger	1.06	1.02, 1.11**
Cynical hostility	1.02	0.97, 1.06
Stressful life events	1.01	0.96, 1.05
Financial strain	1.04	0.98, 1.10
Daily discrimination	1.03	0.99, 1.07
Major discrimination	1.04	0.99, 1.08
**Social Factors**		
Living with spouse/partner	0.90	0.77, 1.05
Contact children		
<Every few months	Reference	Reference
1-2x/month	1.08	0.91, 1.29
1-2x/week	1.05	0.91, 1.21
≥3x/week	1.04	0.87, 1.24
Contact other family		
<Every few months	Reference	Reference
1-2x/month	0.99	0.89, 1.10
1-2x/week	0.92	0.81, 1.05
≥3x/week	0.95	0.85, 1.06
Contact friends		
<Every few months	Reference	Reference
1-2x/month	0.93	0.82, 1.05
1-2x/week	0.94	0.84, 1.04
≥3x/week	0.94	0.83, 1.07
Loneliness	1.05	1.00, 1.11
Closeness with spouse	1.03	0.95, 1.12
Number of close children	1.03	1.00, 1.07
Number of close other family	1.02	0.98, 1.06
Number of close friends	1.03	0.97, 1.10
Positive social support from spouse	0.99	0.91, 1.08
Positive social support from children	1.03	0.99, 1.08
Positive social support from other family	0.99	0.95, 1.04
Positive social support from friends	1.03	0.98, 1.09
Social strain from spouse	1.02	0.93, 1.12
Social strain from children	1.02	0.96, 1.08
Social strain from other family	1.05	1.00, 1.10*
Social strain from friends	1.02	0.97, 1.07
Religious service attendance		
Not at all	Reference	Reference
<1x/week	1.00	0.90, 1.10
≥1x/week	0.93	0.83, 1.05
Volunteering		
0 hours/year	Reference	Reference
1–49 hours/year	1.02	0.90, 1.14
50–99 hours/year	0.92	0.79, 1.07
100–199 hours/year	0.99	0.87, 1.13
≥200 hours/year	0.84	0.70, 1.01
Helping friends/neighbors/relatives		
0 hours/year	Reference	Reference
1–49 hours/year	0.82	0.76, 0.90***
50–99 hours/year	0.85	0.74, 0.98*
100–199 hours/year	0.73	0.63, 0.85***
≥200 hours/year	0.97	0.80, 1.17
Social status ladder	0.99	0.95, 1.04
Change in social status ladder		
Moved down	Reference	Reference
No change	0.94	0.84, 1.04
Moved up	1.00	0.86, 1.17
**Work**		
In labor force	0.90	0.80, 1.02

*p < .05 before Bonferroni correction;

**p < .01 before Bonferroni correction;

***p < .05 after Bonferroni correction (the *p*-value cutoff for Bonferroni correction is *p* = .05/62 predictors: *p* < .00080645).

Abbreviations: CI, confidence interval.

^a^The analytic sample was restricted to those who had participated in the pre-baseline wave (2006/2008). Multiple imputation was performed to impute missing data on the exposures, covariates, and outcome. Candidate predictors were assessed, one at a time, at baseline (2010/2012), and the outcome (number of overnight hospital stays) was assessed during the two years prior to the outcome wave (2012–2014 (Cohort A) or 2014–2016 (Cohort B)). All models adjusted for sociodemographic factors, personality factors, prior values of all candidate predictors, and prior values of the outcome (number of overnight hospital stays), each of which was assessed at pre-baseline (2006/2008).

^b^All continuous candidate predictors were standardized (mean = 0; standard deviation = 1).

^c^An exposure-wide analytic approach was used, and a separate model for each exposure was run. Because the number of overnight hospital stays was a count outcome with a skewed distribution, we ran a generalized linear model with a negative binomial distribution and log link to estimate a rate ratio.

**Table 3:** Candidate predictors of total nights spent in the hospital (Health and Retirement Study [HRS]: N = 11,374) ^a^^,^^b^^,^^c^.

Candidate Predictor	Rate Ratio	95% CI
**Health Behaviors**		
Frequent physical activity	0.73	0.66, 0.81***
Smoking	0.74	0.59, 0.91**
Heavy drinking	1.03	0.79, 1.34
Sleep problems	1.07	0.89, 1.28
**Physical Health**		
Number of physical conditions	1.38	1.26, 1.50***
Diabetes	1.27	1.07, 1.50**
Hypertension	1.09	0.94, 1.26
Stroke	1.42	1.14, 1.79**
Cancer	1.59	1.33, 1.89***
Heart disease	1.62	1.44, 1.82***
Lung disease	1.87	1.54, 2.27***
Arthritis	1.21	1.01, 1.46*
Overweight/obese	0.89	0.79, 1.01
Physical functioning limitations	1.48	1.36, 1.62***
Cognitive impairment	1.07	0.92, 1.23
Chronic pain	1.13	1.02, 1.24*
Self-rated health	0.76	0.73, 0.80***
Hearing	0.98	0.93, 1.04
Eyesight	0.91	0.88, 0.95***
**Psychological Well-being**		
Positive affect	0.95	0.88, 1.02
Life satisfaction	0.91	0.87, 0.95***
Optimism	0.94	0.89, 1.00
Purpose in life	0.93	0.88, 0.97**
Mastery	0.92	0.87, 0.98**
Health mastery	0.86	0.83, 0.91***
Financial mastery	0.98	0.93, 1.03
**Psychological Distress**		
Depression	1.08	0.96, 1.22
Depressive symptoms	1.09	1.05, 1.13***
Hopelessness	1.06	0.99, 1.14
Negative affect	1.07	1.02, 1.13*
Constraints	1.16	1.11, 1.21***
Anxiety	1.15	1.09, 1.22***
Trait anger	1.02	0.95, 1.10
State anger	1.15	1.09, 1.22***
Cynical hostility	1.04	1.00, 1.08
Stressful life events	0.97	0.92, 1.02
Financial strain	1.06	0.97, 1.15
Daily discrimination	1.06	1.02, 1.11**
Major discrimination	0.96	0.91, 1.02
**Social Factors**		
Living with spouse/partner	0.98	0.76, 1.28
Contact children		
<Every few months	Reference	Reference
1-2x/month	0.90	0.76, 1.08
1-2x/week	0.84	0.73, 0.96*
≥3x/week	0.86	0.73, 1.01
Contact other family		
<Every few months	Reference	Reference
1-2x/month	0.83	0.71, 0.96*
1-2x/week	0.83	0.69, 0.99*
≥3x/week	0.77	0.64, 0.94*
Contact friends		
<Every few months	Reference	Reference
1-2x/month	0.96	0.85, 1.08
1-2x/week	0.95	0.84, 1.08
≥3x/week	0.98	0.85, 1.12
Loneliness	1.04	0.99, 1.09
Closeness with spouse	1.08	0.96, 1.21
Number of close children	1.01	0.95, 1.07
Number of close other family	0.98	0.93, 1.02
Number of close friends	1.02	0.96, 1.08
Positive social support from spouse	1.01	0.90, 1.12
Positive social support from children	1.05	1.01, 1.09*
Positive social support from other family	0.95	0.89, 1.01
Positive social support from friends	1.07	0.98, 1.16
Social strain from spouse	0.99	0.88, 1.11
Social strain from children	1.00	0.95, 1.05
Social strain from other family	1.00	0.92, 1.09
Social strain from friends	1.02	0.96, 1.08
Religious service attendance		
Not at all	Reference	Reference
<1x/week	0.93	0.85, 1.02
≥1x/week	0.86	0.78, 0.95**
Volunteering		
0 hours/year	Reference	Reference
1–49 hours/year	0.87	0.78, 0.98*
50–99 hours/year	0.85	0.75, 0.97*
100–199 hours/year	0.77	0.65, 0.92**
≥200 hours/year	0.77	0.64, 0.92**
Helping friends/neighbors/relatives		
0 hours/year	Reference	Reference
1–49 hours/year	0.82	0.75, 0.89***
50–99 hours/year	0.79	0.70, 0.89***
100–199 hours/year	0.72	0.64, 0.82***
≥200 hours/year	0.77	0.64, 0.92**
Social status ladder	0.99	0.92, 1.07
Change in social status ladder		
Moved down	Reference	Reference
No change	0.94	0.80, 1.09
Moved up	0.91	0.70, 1.18
**Work**		
In labor force	1.02	0.90, 1.15

*p < .05 before Bonferroni correction;

**p < .01 before Bonferroni correction;

***p < .05 after Bonferroni correction (the *p*-value cutoff for Bonferroni correction is *p* = .05/62 predictors: *p* < .00080645).

Abbreviations: CI, confidence interval.

^a^The analytic sample was restricted to those who had participated in the pre-baseline wave (2006/2008). Multiple imputation was performed to impute missing data on the exposures, covariates, and outcome. Candidate predictors were assessed, one at a time, at baseline (2010/2012), and the outcome (number of nights spent overnight in the hospital) was assessed during the two years prior to the outcome wave (2012–2014 (Cohort A) or 2014–2016 (Cohort B)). All models adjusted for sociodemographic factors, personality factors, prior values of all candidate predictors, and prior values of the outcome (number of nights spent overnight in the hospital), each of which was assessed at pre-baseline (2006/2008).

^b^All continuous candidate predictors were standardized (mean = 0; standard deviation = 1).

^c^An exposure-wide analytic approach was used, and a separate model for each exposure was run. Because the number of nights spent overnight in the hospital was a count outcome with a skewed distribution, we ran a generalized linear model with a negative binomial distribution and log link to estimate a rate ratio.

When considering health behaviors, two out of four candidate predictors were associated with subsequent hospitalizations. Those engaging in frequent (≥1x/week) physical activity had 0.80 the rate of overnight hospital stays (95% CI: 0.74, 0.87), and who reported current smoking had 0.77 the rate of overnight hospital stays (95% CI: 0.63, 0.93). There was little evidence of associations with other health behaviors (e.g., heavy drinking, and sleep problems). For physical health factors, 11 out of 15 candidate predictors were associated with overnight hospitalizations. The strongest associations were observed among people with lung disease (RR = 1.56; 95% CI: 1.33, 1.83) and cancer (RR = 1.57; 95% CI: 1.35, 1.82).

Nearly half of the psychological factors (9 out of 20) were associated with subsequent hospitalizations. Among psychological well-being factors, life satisfaction (RR = 0.91; 95% CI: 0.87, 0.96), purpose in life (RR = 0.92; 95% CI: 0.87, 0.98), mastery (RR = 0.92; 95% CI: 0.88, 0.97), and health mastery (RR = 0.92; 95% CI: 0.88, 0.96), were associated with fewer overnight hospital stays. Among psychological distress factors, constraints (RR = 1.17; 95% CI: 1.12, 1.23) and anxiety (RR = 1.15; 95% CI: 1.08, 1.23) were most strongly associated with more overnight hospital stays.

Two out of 22 social factors were associated with subsequent overnight hospital stays. Social strain from other family members (RR = 1.05; 95% CI: 1.00, 1.10) was associated with an increased rate of overnight hospital stays, while helping friends/neighbors/relatives (1–49 hours/year (RR = 0.82; 95% CI: 0.76, 0.90), 50–99 hours/year (RR = 0.85; 95% CI: 0.74, 0.98), and 100–199 hours/year (RR = 0.73; 95% CI: 0.63, 0.85)) was associated with a decreased rate of overnight hospital stays. The results for the total nights spent in the hospital were quite different for some social factors. Notably, contact with children (1-2x/week; RR = 0.84; 95% CI: 0.73, 0.96), contact with other family (1-2x/month (RR = 0.83; 95% CI: 0.71, 0.96), 1-2x/week (RR = 0.83; 95% CI: 0.69, 0.99), and ≥3x/week (RR = 0.77; 95% CI: 0.64, 0.94)), volunteering (1–49 hours/year (RR = 0.87; 95% CI: 0.78, 0.98), 50–99 hours/year (RR = 0.85; 95% CI: 0.75, 0.97), 100–199 hours/year (RR = 0.77; 95% CI: 0.65, 0.92), and ≥200 hours/year (RR = 0.77; 95% CI: 0.64, 0.92)) and religious service attendance (≥1x/week; RR = 0.86; 95% CI: 0.78, 0.95) were all associated with a decreased number of nights in the hospital, while positive social support from children (RR = 1.05; 95% CI: 1.01, 1.09) was associated with an increased number of nights spent in the hospital.

### Additional analyses

We conducted several additional analyses. First, E-values suggested that many of the observed associations were moderately robust to unmeasured confounding (Table 4).

**Table 4:** Robustness to unmeasured confounding (E-values) for the associations between candidate antecedents and hospitalizations (N = 11,374)^a^.

	Number of Overnight Hospital Stays		Number of Nights Spent Overnight in the Hospital	
	Effect Estimate^b^	Confidence Interval Limit^c^	Effect Estimate^b^	Confidence Interval Limit^c^
**Health Behaviors**				
Frequent physical activity	1.80	1.57	2.09	1.79
Smoking	1.93	1.35	2.06	1.43
Heavy drinking	1.12	1.00	1.20	1.00
Sleep problems	1.43	1.00	1.34	1.00
**Physical Health**				
Number of physical conditions	2.06	1.86	2.10	1.83
Diabetes	1.62	1.18	1.85	1.34
Hypertension	1.50	1.00	1.41	1.00
Stroke	1.90	1.45	2.20	1.53
Cancer	2.51	2.03	2.55	1.99
Heart disease	2.33	1.96	2.63	1.25
Lung disease	2.50	2.00	3.15	2.45
Arthritis	1.77	1.25	1.72	1.10
Overweight/obese	1.09	1.00	1.48	1.00
Physical functioning limitations	2.16	1.91	2.33	2.05
Cognitive impairment	1.27	1.00	1.33	1.00
Chronic pain	1.57	1.32	1.50	1.16
Self-rated health	1.93	1.79	1.95	1.81
Hearing	1.26	1.00	1.15	1.00
Eyesight	1.35	1.21	1.43	1.30
**Psychological Well-being**				
Positive affect	1.26	1.00	1.29	1.00
Life satisfaction	1.42	1.27	1.44	1.30
Optimism	1.12	1.00	1.31	1.00
Purpose in life	1.39	1.16	1.37	1.20
Mastery	1.38	1.19	1.38	1.18
Health mastery	1.39	1.23	1.58	1.44
Financial mastery	1.12	1.00	1.19	1.00
**Psychological Distress**				
Depression	1.36	1.00	1.37	1.00
Depressive symptoms	1.38	1.22	1.41	1.28
Hopelessness	1.03	1.00	1.32	1.00
Negative affect	1.44	1.30	1.34	1.14
Constraints	1.62	1.48	1.59	1.47
Anxiety	1.57	1.38	1.57	1.40
Trait anger	1.15	1.00	1.17	1.00
State anger	1.32	1.15	1.57	1.41
Cynical hostility	1.14	1.00	1.24	1.00
Stressful life events	1.10	1.00	1.22	1.00
Financial strain	1.24	1.00	1.31	1.00
Daily discrimination	1.21	1.00	1.32	1.15
Major discrimination	1.23	1.00	1.23	1.00
**Social Factors**				
Living with spouse/partner	1.47	1.00	1.15	1.00
Contact children				
<Every few months	Reference	Reference	Reference	Reference
1-2x/month	1.37	1.00	1.46	1.00
1-2x/week	1.27	1.00	1.68	1.26
≥3x/week	1.24	1.00	1.60	1.00
Contact other family				
<Every few months	Reference	Reference	Reference	Reference
1-2x/month	1.11	1.00	1.71	1.26
1-2x/week	1.40	1.00	1.71	1.09
≥3x/week	1.30	1.00	1.90	1.32
Contact friends				
<Every few months	Reference	Reference	Reference	Reference
1-2x/month	1.36	1.00	1.26	1.00
1-2x/week	1.33	1.00	1.27	1.00
≥3x/week	1.33	1.00	1.18	1.00
Loneliness	1.28	1.00	1.24	1.00
Closeness with spouse	1.21	1.00	1.36	1.00
Number of close children	1.22	1.00	1.08	1.00
Number of close other family	1.15	1.00	1.18	1.00
Number of close friends	1.21	1.00	1.14	1.00
Positive social support from spouse	1.11	1.00	1.08	1.00
Positive social support from children	1.22	1.00	1.28	1.10
Positive social support from other family	1.09	1.00	1.29	1.00
Positive social support from friends	1.22	1.00	1.33	1.00
Social strain from spouse	1.17	1.00	1.11	1.00
Social strain from children	1.16	1.00	1.03	1.00
Social strain from other family	1.28	1.05	1.04	1.00
Social strain from friends	1.14	1.00	1.17	1.00
Religious service attendance				
Not at all	Reference	Reference	Reference	Reference
<1x/week	1.06	1.00	1.35	1.00
≥1x/week	1.35	1.00	1.60	1.27
Volunteering				
0 hours/year	Reference	Reference	Reference	Reference
1–49 hours/year	1.14	1.00	1.56	1.17
50–99 hours/year	1.40	1.00	1.62	1.20
100–199 hours/year	1.09	1.00	1.91	1.40
≥200 hours/year	1.67	1.00	1.93	1.38
Helping friends/neighbors/relatives				
0 hours/year	Reference	Reference	Reference	Reference
1–49 hours/year	1.72	1.47	1.75	1.48
50–99 hours/year	1.63	1.19	1.85	1.51
100–199 hours/year	2.08	1.64	2.11	1.75
≥200 hours/year	1.22	1.00	1.92	1.39
Social status ladder	1.09	1.00	1.10	1.00
Change in social status ladder				
Moved down	Reference	Reference	Reference	Reference
No change	1.33	1.00	1.34	1.00
Moved up	1.04	1.00	1.43	1.00
**Work**				
In labor force	1.45	1.00	1.17	1.00

^a^See VanderWeele and Ding (2017) for the formula for calculating E-values.

^b^The E-values for effect estimates are the minimum strength of association on the rate ratio scale that an unmeasured confounder would need to have with both the exposure and the outcome to fully explain away the observed association between the exposure and outcome, conditional on the measured covariates.

^c^The E-values for the limit of the 95% confidence interval (CI) closest to the null denote the minimum strength of association on the rate ratio scale that an unmeasured confounder would need to have with both the exposure and the outcome to shift the confidence interval to include the null value, conditional on the measured covariates.

For example, for lung disease, an unmeasured confounder associated with both the number of overnight hospital stays and lung disease by risk ratios of 2.50 each (above and beyond the covariates already adjusted for) could explain away the association, but weaker joint confounding associations could not. Further, to shift the CI to include the null, an unmeasured confounder associated with both the number of overnight hospital stays and lung disease by risk ratios of 2.00 could suffice, but weaker joint confounding associations could not. Second, our analyses using zero-inflated negative binomial regression (S1 and S2 Tables) and zero-inflated Poisson regression (S3 and S4 Tables) show fewer associations for participants who are not “certain zeros” (i.e., respondents who are not always certain to have values of zero for hospitalization variables) in negative binomial and Poisson regression models (see footnotes of S1–S4 Tables for additional details). Third, results from complete-case analyses and imputed analyses showed somewhat similar results (S5 and S6 Tables).

## Discussion

In a prospective and national sample of U.S. adults aged >50, we examined associations between changes in 62 candidate predictors and subsequent hospitalizations. Two health behaviors (e.g., frequent physical activity, smoking), and some physical health conditions (e.g., heart disease), psychological well-being- (e.g., purpose in life), psychological distress- (e.g., anxiety), and social-factors (e.g., helping friends/neighbors/relatives) were associated with subsequent hospitalizations. However, there was little evidence of associations with other factors. We generally observed the largest effect sizes for physical health conditions. However, many psychosocial factors were also associated with hospitalizations, and some displayed effect sizes of comparable magnitude to physical health conditions and health behaviors. For example, informal helping (helping friends/neighbors/relatives) showed equivalent (or larger) effect sizes than more well-established physical health conditions (e.g., diabetes) and health behaviors (e.g., smoking). Many of these psychosocial factors can be intervened upon, and thus present novel targets for reducing hospitalizations. For example, some recent meta-analyses of randomized controlled trials suggest that some interventions can improve psychological well-being [50–52]. These interventions have used various methods to enhance psychological well-being, including individual or group therapy, expressing gratitude, practicing prosociality, or writing about positive life events [53]. In other work, researchers have intervened on social factors, identifying methods such as cognitive behavioural therapy to address maladaptive social cognition, as well as multi-level systems approach to enhance social relationships [54, 55].

There were some notable differences when comparing our two outcomes and we provide two illustrative examples that both concern social factors. First, contact with other family, contact with children, and religious service attendance (all markers of a strong social network) predicted fewer total nights spent in the hospital, but not the number of separate overnight hospital stays. A strong social network might provide hospitalized patients with several types of support that enable them to recover more quickly and also a place to recover after discharge (e.g., access to resources (e.g., housing, caretakers), social support (e.g., instrumental-, financial-, informational-, emotional-support), social influence (e.g., reinforcing healthy norms that facilitate recovery)) [55]. Along the same lines, social strain from other family was associated with an increased rate of overnight hospital stays, perhaps due to an inability to rely on other family members for support. However, positive social support from children was associated with increased total nights spent in the hospital, potentially due to encouragement of utilization of hospitalization services from children.

Many of our results converged with results from prior studies. For example, increased chronic conditions [5, 6] and lower purpose in life [12] have been associated with increased hospitalizations. However, some of our results diverged from prior studies (e.g., sleep problems were not associated with subsequent hospitalizations in our study [10]). Methodologically, the underlying reasons for diverging results might stem from a variety of sources including differences in: 1) study design (cross-sectional vs. longitudinal), 2) sample composition (our study examined older adults, and many prior studies evaluated specific subpopulations such as low-income public rental flat dwellers in Singapore or cardiovascular disease patients [26, 27]), 3) assessment of exposures (different instruments), 4) covariate control, 5) measurement of the outcome (e.g., other studies sometimes evaluated: hospitalization risk, risk of potentially preventive hospitalizations [11, 18]), 6) key underlying moderators (samples from varying countries with varying healthcare and insurance systems [5, 13, 16, 17, 27, 56, 57]), and 7) adjustment for prior values of the exposures and outcomes. Future studies may benefit from further exploring hypotheses generated from our study findings. For example, amongst psychological well-being factors, life satisfaction, purpose in life, mastery, and health mastery were associated with fewer overnight hospital stays. Future studies may assess the mechanistic pathways underlying why these psychological well-being factors were related to subsequent hospitalizations. Perhaps purpose in life increases adherence to medical treatment, resulting in better health and decreased hospitalizations. With further research, future studies may also intervene upon promising predictors (e.g., purpose in life) in randomized controlled trials and assess subsequent hospitalizations.

Our study had several limitations. First, we used self-reported data. However, self-reported hospitalizations have displayed high agreement with administrative claims and medical records [58–61]. For example, a study conducted in HRS reported that self-reported number of hospital episodes showed high concordance (κ = 0.77) with claims-based data [61]. Further, a study of community-dwelling older adults comparing self-reported hospital visits and length of stay against medical records, observed near-perfect correspondence (weighted κ = 1.0) [62]. Second, there is potential for unmeasured confounding. Although we had robust covariate adjustment and a longitudinal design, the effect sizes were relatively modest and E-value calculations for the confidence intervals suggested that a combination of statistical uncertainty and unmeasured confounding might constitute an alternative explanation to there being a causal effect. The E-values for the estimates themselves were sometimes rather more substantial and a larger sample size might help provide further evidence. Nevertheless, given the robust design, we believe our analyses might be helpful in identifying potential new intervention targets for future research. Further, even modest effect sizes can have a very substantial impact on costs at the population level (especially given the extent of costs for an overnight hospitalization). Third, we lacked information on the reasons for hospitalizations. Future studies might benefit from assessing specific reasons for overnight hospital stays as different predictors might be more, or less, important for different types of hospitalization [56, 63]. Our study also had several strengths, including use of a large, diverse, prospective, and national sample of U.S. adults aged >50 years.

## Conclusion

The rapidly growing cost of healthcare is an important issue from a medical, political, and economic perspective. As the cost of hospitalizations continue escalating [64], researchers and policymakers are seeking potentially modifiable predictors of hospitalizations with increasing urgency. The purpose of our study was to draw attention to specific physical-, behavioral-, and psychosocial-factors that might impact overnight hospitalizations in older adults. We aimed to identify a more comprehensive pool of options that future investigators might consider as determinants of overnight hospitalizations. With further research, intervening on many of the predictors identified in our study might be a novel way of simultaneously decreasing hospitalizations and enhancing the well-being of our rapidly growing older adult population.

## Supporting information

S1 Appendix Assessment of candidate predictors. (DOCX)

S2 Appendix Proof illustrating how adjusting for pre-baseline levels of an exposure can help us evaluate how “changes” in an exposure are associated with subsequent hospitalizations over time. (DOCX)

S1 Table Candidate predictors of overnight hospital stays, zero-inflated negative binomial (Health and Retirement Study [HRS]: N = 11,374). (DOCX)

S2 Table Candidate predictors of total nights spent in the hospital, zero-inflated negative binomial (Health and Retirement Study [HRS]: N = 11,374). (DOCX)

S3 Table Candidate predictors of overnight hospital stays, zero-inflated poisson (Health and Retirement Study [HRS]: N = 11,374). (DOCX)

S4 Table Candidate predictors of total nights spent in the hospital, zero-inflated poisson (Health and Retirement Study [HRS]: N = 11,374). (DOCX)

S5 Table Complete-case analyses: Candidate predictors of overnight hospital stays (Health and Retirement Study [HRS]: N ranged from = 2,995 to 5,389). (DOCX)

S6 Table Complete-case analyses: Candidate predictors of total nights spent in the hospital (Health and Retirement Study [HRS]: N ranged from = 2,976 to 5,359). (DOCX)

S7 Table Changes in hospitalizations from the pre-baseline wave (t0) to the outcome wave (t2). (DOCX)
