# Supplementary material for: Pathways to reduced overnight hospitalizations in older adults: Evaluating 62 physical, behavioral, and psychosocial factors
Source: PLoS One. 2022 Nov 10;17(11):e0277222. doi: 10.1371/journal.pone.0277222 (PMC9648713; doi:10.1371/journal.pone.0277222)
Supplement: S1 Appendix — (DOCX) [file pone.0277222.s001.docx]

Pathways to reduced overnight hospitalizations in older adults: Evaluating 62 physical, behavioral, and psychosocial factors

**S1 APPENDIX**

**Assessment of candidate predictors.**

**Reference group**

The reference group was the healthiest group for all binary predictors unless otherwise noted.

**Health behaviors**

*Frequent physical activity*. Based on prior research, a binary physical activity variable was created: ≥1x/week of vigorous or moderate exercise was considered frequent physical activity, while <1x/week of vigorous or moderate exercise was the reference group [1]. Participants indicated the frequency (i.e., response categories: daily, >1x/week, 1x/week, 1-3x/month, hardly ever or never) with which they engaged in vigorous (e.g., running, swimming, aerobics), moderate (e.g., gardening, dancing, walking at a moderate pace), and light (e.g., vacuuming, laundry) activities over the past 12 months.

*Smoking*. Participants were asked (yes/no), “Do you smoke cigarettes now?” to assess current smoking status. The reference group was “no” smoking.

*Heavy drinking.* Following the National Institute on Alcohol Abuse and Alcoholism guidelines [2], heavy drinking was defined as >14 for drinks/week for men and >7 drinks/week for women. Alcohol consumption was measured by multiplying the number of days/week that alcohol was consumed x the number of drinks/day, which resulted in the number of drinks/week. Participants not in this alcohol consumption range were classified as non-heavy drinkers (the reference group).

*Sleep problems.* Participants completed the 4-item Jenkins Sleep Questionnaire, a widely used and validated screening instrument for assessing sleep complaints and insomnia symptoms [3]. Response categories included “most of the time,” “sometimes,” and “rarely or never.” Having sleep problems was defined as reporting: “most of the time” for any of the three negatively worded items (e.g., “How often do you have trouble falling asleep?”) and “rarely or never” to the one positively worded item (i.e., “feel really rested when you wake up in the morning”). Participants were considered unhealthy (i.e., having sleep problems) if they reported one or more sleep problems. The sleep questionnaire was only administered every other wave. Thus, sleep data was imputed for half of the sample.

**Physical health­­­­**

*Physical conditions.* Participants self-reported (yes/no) if they were ever told by a healthcare provider that they had the following conditions: 1) diabetes, 2) hypertension, 3) stroke, 4) cancer, 5) heart disease, 6) lung disease, or 7) arthritis. The Health and Retirement Study (HRS) has demonstrated validity and reliability of self-reported chronic conditions [4].

*Overweight/obesity*. Body mass index (BMI) was derived from self-reported height and weight. It was calculated as weight/height^2^ (kg/m^2^). A BMI of ≥25 kg/m^2^ was considered as overweight/obese [5].

*Number of physical conditions.* To create a score for the number of physical conditions, a summary score was calculated by summing the number of reported conditions. This measure included the 7 chronic conditions above and overweight/obesity (range 0-8).

*Physical functioning limitations.* Physical functioning limitations were assessed using items from scales developed by Rosow and Breslau (1966), Nagi (1976), Katz, Ford, Moskowitz, Jackson, and Jaffe (1963), and Lawton and Brody (1969) [6–9]. A total of 15 questions about physical functioning (e.g., walking several blocks, climbing one flight of stairs, pushing or pulling large objects, lifting or carrying 10 pounds, getting up from a chair, reaching or extending arms up, stooping, kneeling, or crouching, sitting for 2 hours) and activities of daily living (e.g., walking across a room, dressing, eating, bathing, getting in to or out of bed, using the toilet, picking up a dime) were included. Participants were classified as having “physical functioning limitations” if they reported >4 limitations with physical functioning, while participants who reported <4 limitations were considered “normal” (the reference group). This criterion was determined by identifying the physical function score where 75% of participants could be considered as having healthy physical function in the HRS sample.

*Cognitive impairment*. The HRS cognitive functioning assessment [10,11] was adapted from the modified Telephone Interview for Cognitive Status (TICS-M). The assessment included an immediate and delayed 10-noun free recall test, a serial 7 subtraction test, and a backward count 20 test (27-point scale overall). This assessment tool has been shown to have high sensitivity and specificity when assessing cognitive impairment in older adults. The cut-off points used in this study were derived from previous research on cognitive impairment in HRS [12,13]. Participants who scored 0-11 (on the 27-point scale) were classified as having “cognitive impairment,” while participants who scored ≥12 were classified as “normal” (the reference group). HRS reports contain further information about these cognitive assessments [10,11].

*Chronic pain*. Participants were asked (yes/no), “Are you often troubled with pain?” The reference group was “no” pain.

*Self-rated health.* Participants were asked, “Would you say your health is excellent, very good, good, fair, or poor?” on a 5-point scale (reverse coded with higher scores indicating higher self-rated health).

*Hearing.* Participants were asked, “Is your hearing excellent, very good, good, fair, or poor (using a hearing aid as usual)?” on a 5-point Likert scale (reverse coded with higher values indicating better hearing). Self-report measures of hearing have been found to be reliable measures of hearing impairment [14].

*Eyesight.* Participants were asked, “Is your eyesight excellent, very good, good, fair, or poor using glasses or corrective lenses as usual?” Response categories were as follows: 1) Excellent, 2) Very good, 3) Good, 4) Fair, 5) Poor or 6) Legally blind. Responses were reverse coded such that higher values were associated with better eyesight.

**Psychological well-being**

*Positive affect.* Positive affect was measured (in 2006 only) with a 6-item scale [15–17] originally developed for use in the Midlife in the United States Study. The scale assessed how often the participant felt “cheerful,” “in good spirits,” “extremely happy,” “calm and peaceful,” “satisfied,” and “full of life” over the past 30 days. Response categories ranged from 1 (all of the time) to 5 (none of the time). Responses were reverse scored, so that a higher score indicated higher positive affect. An overall score was derived by averaging responses across all 6 items (α=0.91 in 2006, range=1 to 5). After the 2006 wave, the HRS switched to a more expansive measure of positive affect based on the Positive and Negative Affect Schedule (PANAS-X) [18]. It included the following 13 items: determined, enthusiastic, active, proud, interested, happy, attentive, content, inspired, hopeful, alert, calm, and excited. An overall score was derived by averaging responses across all 13 items (α=0.92 in 2008, range=1 to 5). A limitation of this study is that affect was measured in a different way during only the first wave of the study. However, scores were standardized and both the prior and current measures of affect operate very similarly (e.g., similar correlations with other variables, similar pattern of descriptive statistics).

*Life satisfaction.* Life satisfaction was assessed with the 5-item Satisfaction with Life Scale [19]. The scale has shown excellent psychometric properties in prior work. Using a 7-point Likert scale (from 1 (strongly disagree) to 7 (strongly agree)), participants were asked the extent to which they agreed with statements such as, “In most ways my life is close to ideal.” Responses to all items were averaged to create a composite score, with higher scores indicating higher life satisfaction (α=0.88, range=1 to 7).

*Optimism.* Optimism was assessed with the Life Orientation Test-Revised (LOT-R), which has good discriminant and convergent validity, as well as good reliability [20]. Using a 6-point Likert scale (from 1 (strongly disagree) to 6 (strongly agree)), participants were asked the degree to which they agreed with statements such as, “In uncertain times, I usually expect the best.” Negatively worded items were reverse coded and responses to all items were averaged to create an overall score, with higher scores indicating higher optimism (α=0.75, range=1 to 6).

*Purpose in life.* Purpose in life was assessed with a 7-item purpose in life subscale from Ryff’s Psychological Well-Being Scale [21]. The 7-item subscale has been validated in prior work and has shown good psychometric properties [22]. Using a 6-point Likert scale (from 1 (strongly disagree) to 6 (strongly agree)), participants were asked the degree to which they agreed with statements such as, “I have a sense of direction and purpose in my life.” Negatively worded items were reverse coded and all items were averaged to create a composite score, with higher scores indicating higher purpose (α=0.77, range=1 to 6).

*Mastery.* Mastery was assessed with 5 items derived from Lachman and Weaver (1998). The measure has good discriminant and convergent validity, and good reliability [23]. Using a 6-point Likert scale (from 1 (strongly disagree) to 6 (strongly agree)), participants were asked the degree to which they agreed with statements such as, “I can do just about anything I really set my mind to.” All items were averaged to create a composite score, with higher scores indicating higher mastery (α=0.90, range=1 to 6).

*Health mastery.* Participants were asked, “How would you rate the amount of control you have over your health these days?” on a 0 (“no control at all”) to 10 (“very much control”) scale.

*Financial mastery.* Participants were asked, “How would you rate the amount of control you have over your financial situation these days?” on a 0 (“no control at all”) to 10 (“very much control”) scale.

**Psychological distress**

*Depressive symptoms and depression.* Depressive symptoms were measured using The Center for Epidemiologic Studies Depression Scale (CESD) [24]. This scale has been validated in the HRS [25]. Participants indicated the presence of 8 depressive symptoms (e.g., “Much of the time during the past week, I felt depressed”) over the past week (yes/no). All items were summed, with higher scores indicating higher depressive symptoms (α=0.80, range=0 to 8). Participants with scores of >4 were classified as having depression, as done previously (no depression was the reference group) [25]. Prior work has suggested that the cutoff value of 4 would produce results similar to the 16-item cutoff when using the full (20-item) CESD scale [25].

*Hopelessness.* Hopelessness was assessed with a 4-item questionnaire from two previously validated scales [26,27]. Using a 6-point Likert scale (from 1 (strongly disagree) to 6 (strongly agree)), participants were asked the degree to which they agree with statements such as, “The future seems hopeless to me and I can’t believe that things are changing for the better.” All items were averaged to create a composite score (α=0.86, range=1 to 6), with higher scores indicating more hopelessness.

*Negative affect*. Negative affect was measured (in 2006 only) with a 6-item scale originally developed for use in the Midlife in the United States Study [15–17]. The scale assessed how often the participant felt “so depressed that nothing could cheer you up,” “hopeless,” “restless or fidgety,” “that everything was an effort,” “worthless,” and “nervous” over the past 30 days. Response categories ranged from 1 (all of the time) to 5 (none of the time). Responses were reverse scored, so that a higher score indicated higher negative affect. An overall score was derived by averaging responses across all 6 items (α=0.87, range=1 to 5). After the 2006 wave, the HRS switched to a more expansive measure of negative affect based on the PANAS-X [18]. It included the following 12 items: afraid, upset, guilty, scared, frustrated, bored, hostile, jittery, ashamed, nervous, sad, and distressed. An overall score was derived by averaging responses across all 12 items (α=0.89, range=1 to 5). A limitation of this study is that affect was measured in a different way during only the first wave of the study. However, scores were standardized and both the prior and current measures of affect operate very similarly (e.g., similar correlations with other variables, similar pattern of descriptive statistics).

*Perceived constraints.* Perceived constraints were assessed with 5 other items derived from Lachman and Weaver (1998), and this measure has good discriminant and convergent validity, as well as good reliability [23]. Using a 6-point Likert scale (from 1 (strongly disagree) to 6 (strongly agree)), participants were asked the degree to which they agreed with statements such as, “What happens in my life is often beyond my control.” All items were averaged to create an overall score, with higher scores indicating a higher sense of constraints on personal control (α=0.87, range=1 to 6).

*Anxiety.* Anxiety was assessed using 5 of the 21 items in the Beck Anxiety Inventory (BAI) [28]. This inventory has been shown to differentiate between symptoms of depression and anxiety and has been validated in older adults [29]. Participants were asked, “How often did you feel that way during the past week.” 1) “I had fear of the worst happening,” 2) “I was nervous,” 3) “I felt my hands trembling,” 4) “I had a fear of dying,” and 5) “I felt faint,” and could respond with 1 of 4 categories: 1) Never, 2) Hardly ever, 3) Some of the time, 4) Most of the time. The five responses were averaged, with higher scores indicating greater anxiety symptoms (α=0.81, range=1 to 4).

*Trait anger and state anger.* Trait anger (anger-in) and state anger (anger-out) are the two dimensions along which the Spielberger Anger Expression Scale (STAX) measures anger [30]. These two dimensions have been shown to be separate factors that are modestly correlated through a principal factor analysis with Promax rotation [31]. Trait anger is the predisposition to respond with anger across a variety of situations. To measure this variable, participants were asked to respond to four statements such as, “When I am feeling angry or mad, I keep things in.” State anger is a temporary behavioral reaction of anger and was measured through seven statements including, “When I am feeling angry or mad, I strike out at whatever infuriates me.” Participants gave responses on a 4-point Likert scale for each item: 1) Almost never, 2) Sometimes, 3) Often and 4) Almost always. Responses were averaged for trait anger (α=0.80) and state anger (α=0.82) separately, with higher scores indicating higher trait anger and state anger (range=1 to 4).

*Cynical hostility.* Cynical hostility was measured using 5 items from the Cook-Medley Hostility Inventory [32]. The items were as follows: 1) “Most people dislike putting themselves out to help other people,” 2) “Most people will use somewhat unfair means to gain profit or an advantage rather than lose it,” 3) “No one cares much what happens to you,” 4) “I think most people would lie in order to get ahead,” and 5) “I commonly wonder what hidden reasons another person may have for doing something nice for me.” The first statement was written as, “Most people inwardly dislike putting themselves out to help other people” in the 2006 and 2008 questionnaire before being changed from 2010 onwards. Participants responded on a 6-point Likert scale (from 1 (strongly disagree) to 6 (strongly agree)). The scores were averaged (α=0.78, range=1 to 6), with higher scores indicating higher cynical hostility.

*Stressful life events.* Stressful life events were measured using 5 questions that have been used in other widely-used self-report measures of life stress [33]. Items included questions such as, “Have you been unemployed and looking for work for longer than 3 months at some point in the past five years?” While the questionnaire in 2008 onwards asked an additional question of, “Have you been the victim of fraud in the past five years?”, this was not included for the purposes of the present study to maintain consistency (since it was not included in the 2006 questionnaire). Participants answered each question with a yes or no. Responses (0 = no, 1 = yes) were summed, with higher values indicating a higher number of stressful life events.

*Financial strain.* Respondents were asked, “How difficult is it for (you/your family) to meet monthly payments on (your/your family’s) bills?” and response options included: 1) Not at all difficult, 2) Not very difficult, 3) Somewhat difficult, 4) Very difficult or 5) Completely difficult. Higher scores indicated more financial strain.

*Daily discrimination and major discrimination.* Items measuring daily discrimination and major discrimination were based on prior widely used discrimination assessments [34–36]. Daily discrimination was measured using 5 items that capture the frequency of the following experiences in the day-to-day lives of participants: 1) being treated with less courtesy or respect, 2) receiving poorer service in restaurants or stores, 3) people acting as if you are not smart, 4) people acting as if they are afraid of you, and 5) being threatened or harassed. Participants answered with one of the following response categories: 1) Almost every day, 2) At least once a week, 3) A few times a month, 4) A few times a year, 5) Less than once a year and 6) Never. Items were reverse-coded and averaged (α=0.80, range=1 to 6) such that higher scores indicated higher daily discrimination. The item, “You receive poorer service or treatment than other people from doctors or hospitals” (introduced in 2008) was excluded in the present study to maintain consistency as it was not present in the 2006 questionnaire. Major discrimination was measured using 6 items (yes/no) to capture major instances of lifetime discrimination: 1) being unfairly dismissed from a job, 2) not being hired for a job, 3) being unfairly denied a promotion, 4) being prevented from moving to a neighborhood because the realtor refused to sell/rent to you, 5) being unfairly denied a bank loan, and 6) being unfairly stopped by the police. Responses were summed with higher scores indicating more experiences of major discrimination. One item (“Have you ever been unfairly denied health care or treatment?” (introduced in 2008)) was excluded in the present study to maintain consistency as it was not included in the 2006 questionnaire.

**Social factors**

*Living with a partner/spouse.* Participants were asked, “Do you have a husband, wife, or partner with whom you live?,” and answered yes/no.

*Frequency of contact with children/other family/friends.* The frequency of contact respondents had with members in their social network was evaluated through 3 items each for contacts who had 1) children, 2) other family, and 3) friends. Participants were asked, “On average, how often do you do each of the following?” 1) “Meet up (include both arranged and chance meetings),” 2) “Speak on the phone,” and 3) “Write or email.” Possible response categories were as follows: 1) Three or more times a week, 2) Once or twice a week, 3) Once or twice a month, 4) Every few months, 5) Once or twice a year or 6) Less than once a year or never. The responses were re-coded into the following categories: 0 = Never – every few months, 1 = 1-2x/month, 2 = 1-2x/week and 3 = 3 or more times/week. Participants who reported not having children, other family, or friends were given a value of 0.

*Loneliness.* Loneliness was assessed with three items from the previously validated UCLA Loneliness Scale [37]. Participants were asked, “How much of the time do you feel”: 1) “you lack companionship”, 2) “left out”, and 3) “isolated from others”, with response categories ranging from 1 (often) to 3 (hardly ever or never). Responses were reverse scored and averaged, with higher scores indicating higher loneliness (α=0.80, range=1 to 3).

*Closeness with spouse.* One’s closeness with their spouse, if they had one, was assessed using a single question, “How close is your relationship with your spouse or partner?” Response options included: 1) Very close, 2) Quite close, 3) Not very close and 4) Not at all close. Responses were reverse coded to range from 1 (not at all close) to 4 (very close). To avoid a large drop in sample size for complete-case analyses, responses to the item were recoded into the following categories to include those without a spouse: 0 = not at all close or not very close, 1 = quite close or very close, and 2 = not applicable (does not have a spouse).

*Number of close children, close other family, close friends.* The quantity of close social ties was measured through the following 3 items: 1) “How many of your children would you say you have a close relationship with?”, 2) “How many of these family members would you say you have a close relationship with?”, and 3) “How many of your friends would you say you have a close relationship with?”

*Positive social support from spouse, children, other family, friends + Social strain from spouse, children, other family, friends.* The positive social support and negative social strain associated with each category of social ties were assessed using 3 and 4 items respectively. These items were based on those used in previous studies on social support [38,39]. Items assessing positive social support were as follows: 1) “How much do they really understand the way you feel about things?”, 2) “How much can you rely on them if you have a serious problem?” and 3) “How much can you open up to them if you need to talk about your worries?” The 4 items assessing social strain were: 1) “How often do they make too many demands on you?”, 2) “How much do they criticize you?”, 3) “How much do they let you down when you are counting on them?” and 4) “How much do they get on your nerves?” Response options for all 7 questions included: 1) A lot, 2) Some, 3) A little or 4) Not at all. Scores were reverse coded and then averaged to create separate indexes for positive social support and negative social strain. Higher values indicated more positive social support or more social strain (range=1 to 4). This was done for positive social support from spouse (α=0.80), children (α=0.82), other family (α=0.82), and friends (α=0.84), as well as negative social strain from spouse (α=0.78), children (α=0.77), other family (α=0.78), and friends (α=0.75). In our imputed analyses, participants without a spouse were treated as having missing data (and therefore, data on social support and social strain from a spouse were imputed for these participants). To avoid a large drop in sample size for complete-case analyses, responses were recoded into the following categories to include those without a spouse: 0 = low support/strain from a spouse (average of ≤2.5 on spousal social support/strain items), 1 = high support/strain from a spouse (average of >2.5 on the spousal social support/strain items), and 2 = not applicable (does not have a spouse).

*Religious service attendance.* Participants were asked, “About how often have you attended religious services during the past year?” Possible response categories were as follows: 1) More than once a week, 2) Once a week, 3) Two or three times a month, 4) One or more times a year, or 5) Not at all. Response categories of 1 or 2 were redefined as “>1x/week.” Response categories of 3 or 4 were redefined as “<1x/week.” A response category of 5 was consistently defined as “Not at all.”

*Volunteering.* Respondents were asked, “Have you spent any time in the past 12 months doing volunteer work for religious, educational, health-related or other charitable organizations?” If they answered yes to this question, respondents were asked how many hours they volunteered. Responses were coded as: 0 = 0 hours, 1 = 1-49 hours, 2 = 50-99 hours, 3 = 100-199 hours and 4 = ≥ 200 hours. Higher values indicated a greater amount of time spent volunteering.

*Helping friends/neighbors/relatives.* Respondents were asked, “Have you spent any time in the past 12 months helping friends, neighbors, or relatives who did not live with you and did not pay you for the help?” If they answered yes to this question, respondents were asked how many hours they spent helping. Responses were coded as: 0 = 0 hours, 1 = 1-49 hours, 2 = 50-99 hours, 3 = 100-199 hours and 4 = ≥ 200 hours. Higher values indicated a greater amount of time spent helping others.

*Social status ladder + change in social status ladder.* The MacArthur scale of subjective social status was used to evaluate an individual’s own position on the social ladder [40]. Participants were asked to think of a ladder on which the people at the top were best off and those at the bottom were worst off based on money, education level and job quality (e.g., having one of the best jobs vs. having the worst jobs or no job). The first item asked respondents to place themselves on the ladder (range: 1-10). The second item asked, “Has your position on the ladder changed within the last two years?” Participants could answer 1) Yes, I have moved up, 2) Yes, I have moved down or 3) No, my position has not changed. Responses were re-coded into the following categories: 1 = downward movement, 2 = no change and 3 = upward movement.

**Work**

*In labor force.* Participants were asked, “Are you currently working?” An answer of 1 indicated “yes” while a 5 indicated “no”. Responses were recoded such that 1 = “In labor force” and 0 = “Not in labor force”.

## References

1. Nandi A, Glymour MM, Subramanian SV. Association among socioeconomic status, health behaviors, and all-cause mortality in the United States. Epidemiology. 2014 Mar;25(2):170–7.

2. National Institute on Alcohol Abuse and Alcoholism (NIAAA). Drinking Levels Defined [Internet]. [cited 2019 Mar 31]. Available from: https://www.niaaa.nih.gov/alcohol-health/overview-alcohol-consumption/moderate-binge-drinking

3. Jenkins CD, Stanton BA, Niemcryk SJ, Rose RM. A scale for the estimation of sleep problems in clinical research. J Clin Epidemiol. 1988;41(4):313–21.

4. Fisher GG, Faul JD, Weir DR, Wallace RB. Documentation of chronic disease measures in the Health and Retirement Study (HRS/AHEAD). Ann Arbor (MI): University of Michigan, Survey Research Center; 2005. 103 p.

5. World Health Organization. Physical status: the use and interpretation of anthropometry: report of a WHO expert committee. Geneva: Benteli; 1995. 463 p. Report No.: 854. Available from: https://apps.who.int/iris/bitstream/handle/10665/37003/WHO_TRS_854.pdf

6. Rosow I, Breslau N. A Guttman health scale for the aged. J Gerontol. 1966 Oct;21(4):556–9.

7. Nagi SZ. An epidemiology of disability among adults in the United States. Milbank Mem Fund Q Health Soc. 1976;439–67.

8. Katz S, Ford AB, Moskowitz RW, Jackson BA, Jaffe MW. Studies of illness in the aged: the index of ADL: a standardized measure of biological and psychosocial function. JAMA. 1963 Sep 21;185(12):914–9.

9. Lawton MP, Brody EM. Assessment of older people: self-maintaining and instrumental activities of daily living. Gerontologist. 1969;9(3 Part 1):179–86.

10. Fisher GG, Halimah H, Faul JD, Rogers WL, Weir DR. Health and Retirement Study imputation of cognitive functioning measures: 1992 – 2014. Ann Arbor (MI): University of Michigan, Survey Research Center; 2017. 35 p.

11. Ofstedal MB, Fisher GG, Herzog AR. Documentation of cognitive functioning measures in the Health and Retirement Study. Ann Arbor (MI): University of Michigan, Survey Research Center; 2005. 79 p.

12. Crimmins EM, Kim JK, Langa KM, Weir DR. Assessment of cognition using surveys and neuropsychological assessment: the Health and Retirement Study and the Aging, Demographics, and Memory Study. J Gerontol B Psychol Sci Soc Sci. 2011 Jul;(66 Suppl 1):i162-171.

13. Langa KM, Plassman BL, Wallace RB, Herzog AR, Heeringa SG, Ofstedal MB, et al. The Aging, Demographics, and Memory Study: study design and methods. Neuroepidemiology. 2005;25(4):181–91.

14. Chou R, Dana T, Bougatsos C, Fleming C, Beil T. Screening adults aged 50 years or older for hearing loss: a review of the evidence for the U.S. preventive services task force. Ann Intern Med. 2011 Mar 1;154(5):347–55.

15. Watson D, Clark LA, Tellegen A. Development and validation of brief measures of positive and negative affect: the PANAS scales. J Pers Soc Psychol. 1988 Jun;54(6):1063–70.

16. Brim OG, Featherman DL. Surveying midlife development in the United States. Published online 1998.

17. Mroczek DK, Kolarz CM. The effect of age on positive and negative affect: a developmental perspective on happiness. J Pers Soc Psychol. 1998 Nov;75(5):1333–49.

18. Watson D, Clark LA. The PANAS-X: manual for the positive and negative affect schedule-expanded form. 1994. doi: 10.17077/48vt-m4t2

19. Diener E, Emmons RA, Larsen RJ, Griffin S. The Satisfaction With Life Scale. J Pers Assess. 1985 Feb;49(1):71–5.

20. Scheier MF, Carver CS, Bridges MW. Distinguishing optimism from neuroticism (and trait anxiety, self-mastery, and self-esteem): A reevaluation of the Life Orientation Test. J Pers Soc Psychol. 1994 Dec;67(6):1063–78.

21. Ryff CD, Keyes CLM. The structure of psychological well-being revisited. J Pers Soc Psychol. 1995 Oct;69(4):719–27.

22. Abbott R, Ploubidis G, Huppert F, Kuh D, Wadsworth M, Croudace T. Psychometric evaluation and predictive validity of Ryff’s psychological well-being items in a UK birth cohort sample of women. Health Qual Life Outcomes. 2006 Oct 4;(4):76.

23. Lachman ME, Weaver SL. The sense of control as a moderator of social class differences in health and well-being. J Pers Soc Psychol. 1998 Mar;74(3):763–73.

24. Radloff LS. The CES-D Scale: a self-report depression scale for research in the general population. Appl Psychol Meas. 1977 Jun 1;1(3):385–401.

25. Steffeck D. Documentation of affective functioning measures in the Health and Retirement Study. Ann Arbor (MI): University of Michigan, Institute for Social Research; 2000. Available from: https://hrs.isr.umich.edu/sites/default/files/biblio/dr-005.pdf

26. Beck AT, Weissman A, Lester D, Trexler L. The measurement of pessimism: the Hopelessness Scale. J Consult Clin Psychol. 1974 Dec;42(6):861–5.

27. Everson SA, Kaplan GA, Goldberg DE, Salonen R, Salonen JT. Hopelessness and 4-Year progression of carotid atherosclerosis : the Kuopio Ischemic Heart Disease Risk Factor Study. Arterioscler Thromb Vasc Biol. 1997 Aug;17(8):1490–5.

28. Beck AT, Epstein N, Brown G, Steer RA. An inventory for measuring clinical anxiety: psychometric properties. J Consult Clin Psychol. 1988 Dec;56(6):893–7.

29. Wetherell JL, Areán PA. Psychometric evaluation of the Beck Anxiety Inventory with older medical patients. Psychol Assess. 1997 Jun;9(2):136–44.

30. Forgays DK, Spielberger CD, Ottaway SA, Forgays DG. Factor structure of the State-Trait Anger Expression Inventory for middle-aged men and women. Assessment. 1998 Jun;5(2):141–55.

31. Lee Y, Bierman A. A longitudinal assessment of perceived discrimination and maladaptive expressions of anger among older adults: does subjective social power buffer the association? J Gerontol Ser B. 2018 Oct 10;73(8):e120–30.

32. Cook WW, Medley DM. Proposed hostility and Pharisaic-virtue scales for the MMPI. J Appl Psychol. 1954;38(6):414–8.

33. Turner RJ, Wheaton B, Lloyd DA. The epidemiology of social stress. Am Sociol Rev. 1995 Feb 1;60(1):104–25.

34. Williams DR, Yu Y, Jackson JS, Anderson NB. Racial differences in physical and mental health: socio-economic status, stress and discrimination. J Health Psychol. 1997 Jul;2(3):335–51.

35. Essed P. Understanding everyday racism: an interdisciplinary theory [Internet]. Thousand Oaks, California; 1991 [cited 2021 Jan 31]. Available from: https://sk.sagepub.com/books/understanding-everyday-racism-an-interdisciplinary-theory

36. Feagin JR. The continuing significance of race: antiblack discrimination in public places. Am Sociol Rev. 1991 Feb;56(1):101–16.

37. Russell DW. UCLA Loneliness Scale (Version 3): reliability, validity, and factor structure. J Pers Assess. 1996 Feb;66(1):20–40.

38. Schuster TL, Kessler RC, Aseltine RH. Supportive interactions, negative interactions, and depressed mood. Am J Community Psychol. 1990 Jun;18(3):423–38.

39. Turner RJ, Frankel BG, Levin DM. Social support: conceptualization, measurement, and implications for mental health. Res Community Ment Health. 1983;(3):67–111.

40. Adler NE. The MacArthur Scale of Subjective Social Status. [Internet]. [cited 2021 Jan 31]. Available from: https://macses.ucsf.edu/research/psychosocial/subjective.php
