# Supplementary material for: Pathways to reduced overnight hospitalizations in older adults: Evaluating 62 physical, behavioral, and psychosocial factors
Source: PLoS One. 2022 Nov 10;17(11):e0277222. doi: 10.1371/journal.pone.0277222 (PMC9648713; doi:10.1371/journal.pone.0277222)
Supplement: S2 Appendix — (DOCX) [file pone.0277222.s002.docx]

Pathways to reduced overnight hospitalizations in older adults: Evaluating 62 physical, behavioral, and psychosocial factors

**S2 APPENDIX**

# Proof illustrating how adjusting for pre-baseline levels of an exposure can help us evaluate how “changes” in an exposure are associated with subsequent hospitalizations over time.

Let Y be the outcome during the two years prior to t_2_:2014/2016, A_1_ the exposure under consideration in 2010/2012, A_0_ the prior level of exposure in 2006/2008, and C the set of all other covariates in 2006/2008.

For a continuous outcome, the regression model is: E[Y|a_0_, a_1_, c] = v + b_0_a_0_ + b_1_a_1_+ b_2_’c. Let Y_a_ denote the potential outcome Y for an individual under an intervention to set A_1_ to a. For an individual with baseline exposure A_0_=a_0_ and covariates c in 2006/2008, under the no-confounding (and positivity and consistency) and modeling assumptions, a change in exposure of d points A_0_=a_0_ to A_1_=a_0_+d in 2010/2012, rather than maintaining exposure of A_1_=a_0_ in 2010/2012, will give rise to an effect (a difference in potential outcomes for Y) of:

E[Y_a0+d_| A_0_=a_0_, c] - E[Y_a0_| A_0_=a_0_, c]

= E[Y_a0+d_| A_1_=a_0_+d, A_0_=a_0_, c] - E[Y_a0_| A_1_=a_0_, A_0_=a_0_, c]

= E[Y| A_1_=a_0_+d, A_0_=a_0_, c] - E[Y| A_1_=a_0_, A_0_=a_0_, c]

= [v + b_0_a_0_ + b_1_(a_0_+d) + b_2_’c] - [v + b_0_a_0_ + b_1_a_0_ + b_2_’c]

= b_1_d

where the first equality follows by the no-confounding assumption, the second by consistency, and the third by the statistical model.

For a binary outcome, the regression model is: log{P[Y=1|a_0_, a_1_, c]} = v + b_0_a_0_ + b_1_a_1_+ b_2_’c, which, if the outcome is rare can be approximated by a logistic regression model, logit{P[Y=1|a_0_, a_1_, c]} = v + b_0_a_0_ + b_1_a_1_+ b_2_’c, or, if the outcome is common, by a modified Poisson model. Let Y_a_ denote the potential outcome Y for an individual under an intervention to set A_1_ to a. For an individual with baseline exposure A_0_=a_0_ and covariates c in 2006/2008, under the no-confounding (and positivity and consistency) and modeling assumptions, a change in exposure of d points A_0_=a_0_ to A_1_=a_0_+d in 2010/2012, rather than maintaining exposure of A_1_=a_0_ in 2010/2012, will give rise to an effect on the risk ratio scale of:

P[Y_a0+d_=1| A_0_=a_0_, c] / P[Y_a0_=1| A_0_=a_0_, c]

= P[Y_a0+d_=1| A_1_=a_0_+d, A_0_=a_0_, c] / P[Y_a0_=1| A_1_=a_0_, A_0_=a_0_, c]

= P[Y=1| A_1_=a_0_+d, A_0_=a_0_, c] / P[Y=1| A_1_=a_0_, A_0_=a_0_, c]

= exp[v + b_0_a_0_ + b_1_(a_0_+d) + b_2_’c] / exp[v + b_0_a_0_ + b_1_a_0_ + b_2_’c]

= exp(b_1_d)

where the first equality follows by the no-confounding assumption, the second by consistency, and the third by the statistical model.

For a count outcome, a Poisson regression model can be written as: log{E[Y|a_0_, a_1_, c]} = v + b_0_a_0_ + b_1_a_1_+ b_2_’c. Let Y_a_ denote the potential outcome Y for an individual under an intervention to set A_1_ to a. For an individual with baseline exposure A_0_=a_0_ and covariates c in 2006/2008, under the no-confounding (and positivity and consistency) and modeling assumptions, a change in exposure of d points A_0_=a_0_ to A_1_=a_0_+d in 2010/2012, rather than maintaining exposure of A_1_=a_0_ in 2010/2012, will give rise to an effect of:

E[Y_a0+d_| A_0_=a_0_, c] / E[Y_a0_| A_0_=a_0_, c]

= E[Y_a0+d_| A_1_=a_0_+d, A_0_=a_0_, c] / E[Y_a0_| A_1_=a_0_, A_0_=a_0_, c]

= E[Y| A_1_=a_0_+d, A_0_=a_0_, c] / E[Y| A_1_=a_0_, A_0_=a_0_, c]

= exp[v + b_0_a_0_ + b_1_(a_0_+d) + b_2_’c] / exp[v + b_0_a_0_ + b_1_a_0_ + b_2_’c]

= exp(b_1_d)

where the first equality follows by the no-confounding assumption, the second by consistency, and the third by the statistical model.
