# Supplementary material for: Pathways to reduced overnight hospitalizations in older adults: Evaluating 62 physical, behavioral, and psychosocial factors
Source: PLoS One. 2022 Nov 10;17(11):e0277222. doi: 10.1371/journal.pone.0277222 (PMC9648713; doi:10.1371/journal.pone.0277222)
Supplement: S5 Table — (DOCX) [file pone.0277222.s007.docx]

Pathways to reduced overnight hospitalizations in older adults: Evaluating 62 physical, behavioral, and psychosocial factors

**S5 TABLE**

**S5 Table.** **Complete-case analyses: candidate predictors of overnight hospital stays (Health and Retirement Study [HRS]: N ranged from = 2,995 to 5,389).^a,b,c^**

| **Candidate Predictor** | **Rate Ratio** | **95% CI** |
| --- | --- | --- |
| **Health Behaviors** |  |  |
| Frequent physical activity | 0.77 | 0.69, 0.86*** |
| Smoking | 0.95 | 0.73, 1.25 |
| Binge drinking | 1.21 | 0.93, 1.58 |
| Sleep problems | 1.14 | 0.98, 1.32 |
| **Physical Health** |  |  |
| Number of physical conditions | 1.41 | 1.28, 1.56*** |
| Diabetes | 1.19 | 0.97, 1.45 |
| Hypertension | 1.20 | 0.99, 1.45 |
| Stroke | 1.31 | 1.01, 1.70* |
| Cancer | 1.43 | 1.15, 1.77** |
| Heart disease | 1.52 | 1.27, 1.81*** |
| Lung disease | 1.46 | 1.11, 1.91** |
| Arthritis | 1.36 | 1.11, 1.68** |
| Overweight/obese | 1.07 | 0.92, 1.26 |
| Physical functioning limitations | 1.37 | 1.20, 1.57*** |
| Cognitive impairment | 1.24 | 1.08, 1.43** |
| Chronic pain | 1.24 | 1.11, 1.39*** |
| Self-rated health | 0.74 | 0.69, 0.79*** |
| Hearing | 0.97 | 0.90, 1.03 |
| Eyesight | 0.88 | 0.83, 0.93*** |
| **Psychological Well-being** |  |  |
| Positive affect | 0.93 | 0.87, 0.99* |
| Life satisfaction | 0.95 | 0.89, 1.01 |
| Optimism | 0.93 | 0.87, 1.00* |
| Purpose in life | 0.92 | 0.86, 0.98* |
| Mastery | 0.94 | 0.88, 0.99* |
| Health mastery | 0.93 | 0.87, 0.99* |
| Financial mastery | 1.01 | 0.95, 1.08 |
| **Psychological Distress** |  |  |
| Depression | 1.15 | 0.97, 1.36 |
| Depressive symptoms | 1.10 | 1.04, 1.17** |
| Hopelessness | 1.04 | 0.97, 1.11 |
| Negative affect | 1.04 | 0.97, 1.11 |
| Constraints | 1.17 | 1.10, 1.25*** |
| Anxiety | 1.13 | 1.05, 1.21*** |
| Trait anger | 0.95 | 0.89, 1.01 |
| State anger | 1.06 | 0.99, 1.13 |
| Cynical hostility | 1.07 | 1.00, 1.14* |
| Stressful life events | 0.99 | 0.92, 1.05 |
| Financial strain | 1.01 | 0.94, 1.07 |
| Daily discrimination | 1.07 | 1.01, 1.14* |
| Major discrimination | 0.99 | 0.93, 1.06 |
| **Social Factors** |  |  |
| Living with spouse/partner | 0.94 | 0.77, 1.15 |
| Contact children |  |  |
| <Every few months | Reference | Reference |
| 1-2x/month | 1.08 | 0.83, 1.40 |
| 1-2x/week | 1.13 | 0.88, 1.45 |
| >3x/week | 0.95 | 0.74, 1.22 |
| Contact other family |  |  |
| <Every few months | Reference | Reference |
| 1-2x/month | 1.12 | 0.96, 1.31 |
| 1-2x/week | 0.90 | 0.77, 1.05 |
| >3x/week | 1.01 | 0.85, 1.20 |
| Contact friends |  |  |
| <Every few months | Reference | Reference |
| 1-2x/month | 0.99 | 0.82, 1.20 |
| 1-2x/week | 0.97 | 0.81, 1.17 |
| >3x/week | 1.03 | 0.85, 1.25 |
| Loneliness | 1.12 | 1.05, 1.20*** |
| Closeness with spouse | 0.99 | 0.92, 1.08 |
| Number of close children | 1.01 | 0.97, 1.06 |
| Number of close other family | 0.99 | 0.94, 1.04 |
| Number of close friends | 1.02 | 0.96, 1.07 |
| Positive social support from spouse | 0.94 | 0.87, 1.01 |
| Positive social support from children | 1.01 | 0.95, 1.09 |
| Positive social support from other family | 0.96 | 0.90, 1.03 |
| Positive social support from friends | 1.01 | 0.95, 1.08 |
| Social strain from spouse | 1.06 | 0.98, 1.14 |
| Social strain from children | 1.01 | 0.95, 1.08 |
| Social strain from other family | 1.05 | 0.99, 1.12 |
| Social strain from friends | 1.04 | 0.98, 1.11 |
| Religious service attendance |  |  |
| Not at all | Reference | Reference |
| <1x/week | 1.17 | 1.00, 1.36* |
| >1x/week | 1.06 | 0.88, 1.27 |
| Volunteering |  |  |
| 0 hours/year | Reference | Reference |
| 1-49 hours/year | 1.12 | 0.96, 1.30 |
| 50-99 hours/year | 0.93 | 0.78, 1.13 |
| 100-199 hours/year | 1.16 | 0.97, 1.39 |
| ≥200 hours/year | 0.88 | 0.70, 1.10 |
| Helping friends/neighbors/relatives |  |  |
| 0 hours/year | Reference | Reference |
| 1-49 hours/year | 0.84 | 0.75, 0.96** |
| 50-99 hours/year | 0.83 | 0.71, 0.98* |
| 100-199 hours/year | 0.59 | 0.47, 0.72*** |
| >200 hours/year | 1.08 | 0.85, 1.35 |
| Social status ladder | 1.09 | 1.01, 1.17* |
| Change in social status ladder |  |  |
| Moved down | Reference | Reference |
| No change | 1.08 | 0.91, 1.28 |
| Moved up | 1.23 | 0.97, 1.56 |
| **Work** |  |  |
| In labor force | 1.04 | 0.90, 1.20 |

**p* < .05 before Bonferroni correction; ***p* < .01 before Bonferroni correction; ****p* < .05 after Bonferroni correction (the *p*-value cutoff for Bonferroni correction is *p* = .05/62 predictors: *p* < .00080645).

Abbreviations: CI, confidence interval.

^a^The analytic sample was restricted to those who had participated in the pre-baseline wave (2006/2008). Candidate predictors were assessed, one at a time, at baseline (2010/2012), and the outcome (number of overnight hospital stays) was assessed during the two years prior to the outcome wave (2012-2014 (Cohort A) or 2014-2016 (Cohort B)). All models adjusted for sociodemographic factors, personality factors, prior values of all candidate predictors, and prior values of the outcome (number of overnight hospital stays), each of which was assessed at pre-baseline (2006/2008).

^b^All continuous candidate predictors were standardized (mean = 0; standard deviation = 1).

^c^An exposure-wide analytic approach was used, and a separate model for each exposure was run. Because the number of nights spent overnight in the hospital was a count outcome with a skewed distribution, we ran a generalized linear model with a negative binomial distribution and log link to estimate a rate ratio.
