# Supplementary material for: Pathways to reduced overnight hospitalizations in older adults: Evaluating 62 physical, behavioral, and psychosocial factors
Source: PLoS One. 2022 Nov 10;17(11):e0277222. doi: 10.1371/journal.pone.0277222 (PMC9648713; doi:10.1371/journal.pone.0277222)
Supplement: S7 Table — (DOCX) [file pone.0277222.s009.docx]

Pathways to reduced overnight hospitalizations in older adults: Evaluating 62 physical, behavioral, and psychosocial factors

**S7 TABLE**

**S7 Table.** **Changes in hospitalizations from the pre-baseline wave (t_0_) to the outcome wave (t_2_).**

| **Outcomes** | **Pre-Baseline Wave (t_0_)** | **Outcome Wave (t_2_)** |
| --- | --- | --- |
|  | **Mean (SD)** | **Mean (SD)** |
| Number of nights | 1.81 (7.4) | 4.09 (15.2) |
| Number of stays | 0.39 (0.9) | 0.75 (1.7) |
